# Forgotten Ureteral Double-J Stent Complicated by Severe Encrustation in the Bladder

**DOI:** 10.5334/jbr-btr.1179

**Published:** 2016-09-21

**Authors:** Bruno Coulier, Grégory Lefebvre

**Affiliations:** 1 Clinique Saint-Luc, Bouge, BE

**Keywords:** Double-J stent, Encrustation

## Case Report

**Figure 1:**
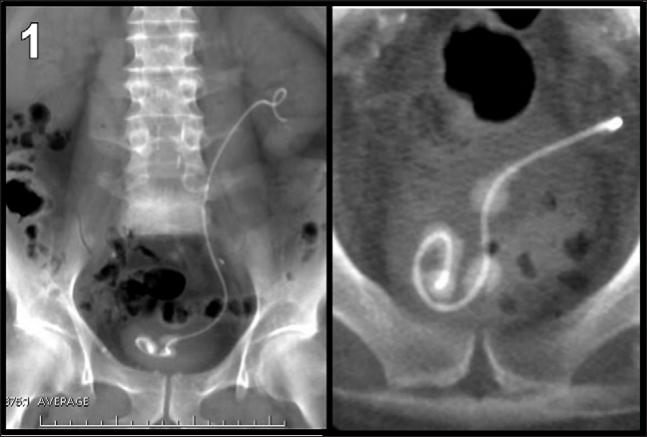


**Figure 2:**
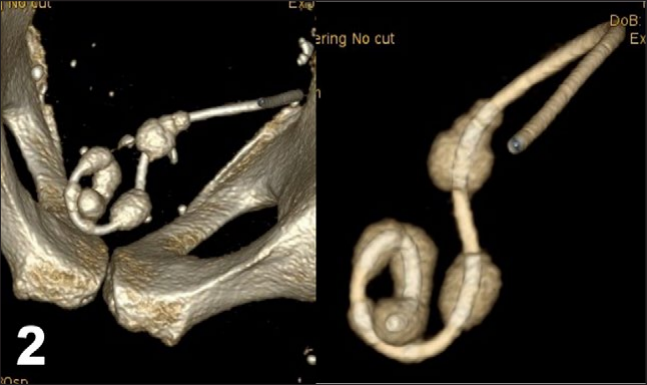


**Figure 3:**
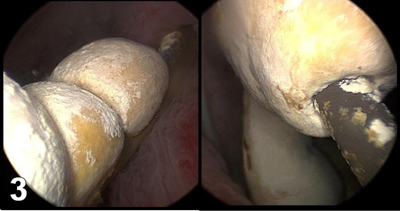


A 58-year-old man was referred to our medical imaging department with complaints of urinary frequency and dysuria associated with suprapubic and hypogastric pain. There was a clinical suspicion of colonic diverticulitis. An unenhanced abdominal CT was performed because the patient’s renal function was unknown. Numerous centimetric stones were found in the bladder, and various volume-rendering reconstructions (Figures 1 and 2) confirmed that these stones had developed like beads on a necklace along the distal intravesical pigtail loop of a double-J ureteral stent. Fortunately, the rest of the stent appeared free from encrustation. Anamnesis of the patient revealed a previous history of a ureteral stone treated with a double-J stent three years earlier in another institution. The diagnosis of encrustation of a forgotten stent was confirmed when the patient underwent a successful cystolithotripsy (Figure 3).

## Comment

Short-term (4 to 6 weeks) placement of a double-J ureteral stent is a very common procedure in daily urologic practice. The indications include the relief of ureteral obstruction secondary to diverse etiologies, accommodating adequate postoperative drainage, and preventing ureteral injuries during surgical procedures.

Mild transitory complications may be present even in a classical short-term use. The typical ‘stent syndrome’ may associate flank pain, frequency, urgency, suprapubic discomfort or pain, and sometime hematuria or incontinence. It is transitory but may cause high morbidity. Alpha-blockers have been used with good results for treating these temporary symptoms.

Most serious long-term complications are associated with prolonged (superior to 6 months) indwelling times [1]. Infection, breakage, malposition or migration of the stent, and stone encrustations were reported. To prevent these events, stents require monitoring while they are in place, removal at the earliest appropriate time, and periodic exchange when chronically indwelling. Risk factors for complications should be minimized with high fluid intake, timely evaluation of clinical complaints, and aggressive treatment of documented infection. Abdominal plain films and ultrasonography are the most common imaging methods for the follow-up, even though our patient underwent CT first because colonic diverticulitis was suspected. Forgotten or overlooked stents – particularly in poor compliant patients – may sometimes irreversibly compromise the renal function.

Stone encrustation, as reported here, is a typical complication of long-term use of double-J stents. Every type of stent represents a foreign body and provides a framework for the deposition of urine constituents, and, over time, encrustation will inevitably occur. Polyurethane stents are more rigid and more prone to encrustation than silicone stents that are more resistant to biofilm formation and thus to secondary encrustation.

Encrustations are more common when stents are used to treat stone disease. They are composed of calcium oxalate, calcium phosphate, and ammonium magnesium phosphate. Predisposed patients are those presenting with a lithogenic history, uricosuria, chronic renal failure, congenital anomalies, urinary tract infection, stasis, dehydration, and long indwelling times. Periodic stent replacement is the only effective method of preventing encrustation.

The size of the stones and the site of encrustation determine the specific therapeutic approach. Because our patient had an exclusively intravesical stone encrustation, he was successfully treated with mechanical cystolithotripsy alone. In more complicated cases in which diffuse encrustation is found from the kidney to the bladder, a combination of procedures may be necessary comprising extracorporeal shock wave lithotripsy (ESWL), percutaneous nephrolithotomy (PCNL), cystolithotripsy (CLT), and ureteroscopic lithotripsy (URSL).
